# Time resolved and label free monitoring of extracellular metabolites by surface enhanced Raman spectroscopy

**DOI:** 10.1371/journal.pone.0175581

**Published:** 2017-04-18

**Authors:** Victoria Shalabaeva, Laura Lovato, Rosanna La Rocca, Gabriele C. Messina, Michele Dipalo, Ermanno Miele, Michela Perrone, Francesco Gentile, Francesco De Angelis

**Affiliations:** 1 Plasmon Nanotechnologies, Istituto Italiano di Tecnologia, Genoa, Italy; 2 Department of Electrical Engineering and Information Technologies (DIETI), University Federico II of Naples, Naples, Italy; Institute of Materials Science, GERMANY

## Abstract

Metabolomics is an emerging field of cell biology that aims at the comprehensive identification of metabolite levels in biological fluids or cells in a specific functional state. Currently, the major tools for determining metabolite concentrations are mass spectrometry coupled with chromatographic techniques and nuclear magnetic resonance, which are expensive, time consuming and destructive for the samples. Here, we report a time resolved approach to monitor metabolite dynamics in cell cultures, based on Surface Enhanced Raman Scattering (SERS). This method is label-free, easy to use and provides the opportunity to simultaneously study a broad range of molecules, without the need to process the biological samples. As proof of concept, NIH/3T3 cells were cultured *in vitro*, and the extracellular medium was collected at different time points to be analyzed with our engineered SERS substrates. By identifying individual peaks of the Raman spectra, we showed the simultaneous detection of several components of the conditioned medium, such as L-tyrosine, L-tryptophan, glycine, L-phenylalanine, L-histidine and fetal bovine serum proteins, as well as their intensity changes during time. Furthermore, analyzing the whole Raman data set with the Principal Component Analysis (PCA), we demonstrated that the Raman spectra collected at different days of culture and clustered by similarity, described a well-defined trajectory in the principal component plot. This approach was then utilized to determine indirectly the functional state of the macrophage cell line Raw 264.7, stimulated with the lipopolysaccharide (LPS) for 24 hours. The collected spectra at different time points, clustered by the PCA analysis, followed a well-defined trajectory, corresponding to the functional change of cells toward the activated pro-inflammatory state induced by the LPS. This study suggests that our engineered SERS surfaces can be used as a versatile tool both for the characterization of cell culture conditions and the functional state of cells over time.

## Introduction

Metabolomics represents the global quantitative analysis of all metabolites in a biological system [1–3]. Types and levels of metabolites produced by living cells can change, depending not only on cell type, but also on their nutritional state, their functional state (i.e. resting or activated cells), and/or their phase of differentiation (i.e. undifferentiated or differentiated stem/precursor cells) [4–6]. On the other hand, different biological molecules, such as amino acids, sugars, proteins and salts, are required at a well-defined concentrations in order to allow specific metabolic processes, thus guaranteeing the optimal viability, as well as specific fate and functions of cells [7–9]. Indeed, wrong or poorly optimized feeding strategies may result in detrimental reduction of nutrients, accumulation of catabolites, lowering in cell growth rate, and functional changes of cells. These events, sometimes hardly identifiable, may result in misleading or inconsistent experiments. Thus, robust tools able to properly monitor cell culture conditions over time are strongly needed, in order to optimize culture strategies and to describe possible functional changes of cells.

Currently, investigations of complex biological samples are mainly performed by nuclear magnetic resonance (NMR) spectroscopy and mass spectrometry (MS) [10–12]. These techniques offer simultaneous structural and quantitative information, with the capability to identify very complex mixtures. However, NMR and MS measurements are destructive for the biological samples, and thus not suitable in such cases when only small amounts of samples are available. For this reason, these techniques do not allow long term monitoring of analytes in live cells, which could represent an important upgrade when working with large-scale culture systems, i.e. cell bioreactors [13].

An attractive alternative to such methods to analyse complex biological samples is represented by microfluidic platforms integrated with surface enhanced optical spectroscopies techniques [14–15]. These tools are simple and relatively inexpensive, and they could become the routine methods for time resolved monitoring of analytes of interest [16–18]. Among plasmonic assisted techniques for biosensing, the most promising candidate is probably Raman spectroscopy, which is label-free, non-destructive and relatively fast to allow real time monitoring of analytes [19–20]. As water provides only very weak Raman signals, analysis of aqueous biological samples through this technique is relatively simple, thus enabling the investigations of molecules related to biochemical processes occurring in the intracellular or extracellular compartments [21–27]. On the other hand, Raman scattering presents relatively low cross-sections (in the order of 10^−30^ cm^2^), which implies relatively poor sensitivity. This limitation can be easily overcome by the use of noble metal nanoparticles [28–30] or nanostructured surfaces [31–36], in which the localized electromagnetic field allows a magnification of the intensity of Raman scattering signals, leading to an increased sensitivity of about seven folds. So far, this effect, called surface enhanced Raman scattering (SERS), has been widely explored for the analysis of biological samples for different purposes, i.e. classification of different bacterial species [37], gene expression estimation [38] and detection of drugs [39–40]. Unfortunately, these studies are limited to the analysis of few biomolecules.

In this work, we present a time resolved, label free and non-destructive SERS-based methodology for monitoring the extracellular medium composition and, indirectly, the functional state of cells. The approach is based on cheap and easy-to-use nanostructured silver (Ag) surfaces placed in the cell culture medium. We monitored the culture conditions of NIH/3T3 cells by collecting Raman spectra of the conditioned culture medium over a period of four days. By analysing the intensity of individual Raman peaks, we detected changes over time for several cell medium components, such as L-tyrosine, L-tryptophan, glycine, L-phenylalanine, L-histidine and fetal bovine serum (FBS) proteins. Analysing the whole data set through the Principal Component Analysis (PCA), we highlighted a well-defined trend of the clustered Raman spectra, reflecting the overall variations observed in the Raman spectra over time. Moreover, in order to demonstrate the application of our strategy to the indirect detection of changes in the functional state of cells, we performed the SERS-based analysis on the conditioned medium of the Raw 264.7 cell line stimulated with LPS toward the so-called “activated” inflammatory state. The switch from the “quiescent” to the “activated” state of the Raw 264.7 cells was monitored through the quantification, by ELISA, of the production of the pro-inflammatory cytokine IL12p40, at different time points within 24 hours. Analysing the Raman spectra of the corresponding conditioned media with the PCA analysis, we highlighted that the spectra collected at the different time points clusterized, describing a precise trajectory in the PC plot. This distribution of the clustered Raman spectra reflected the progressive functional change of the cells in a quiescent state toward a pro-inflammatory “activated” state.

## Materials and methods

### Silver island film fabrication

Silver films were prepared through electroless deposition of Ag on silicon substrates [41–42]. In detail, silicon substrates (1x1 cm^2^) were immersed for two minutes in a solution composed by AgNO_3_ 1 mM (SIGMA Aldrich, St. Louis, MO, USA) and HF 0.15 M (Sigma-Aldrich, St. Louis, MO, USA) at 50°C stirred vigorously at 100 rpm. Then, the sample was immediately rinsed twice with Milli-Q water and gently dried under nitrogen flow.

### Cell cultures and ELISA assay

NIH/3T3, mouse embryonic fibroblasts cell line, and Raw 264.7, mouse blood macrophages cell line, were obtained from the American Type Culture Collection (ATCC) and Sigma-Aldrich (St. Louis, MO, USA), respectively. The cells were cultured in Dulbecco's modified Eagle's medium (DMEM) with high glucose content, supplemented with 10% FBS, penicillin G (100 U/mL) and streptomycin sulphate (100 g/L). All reagents were obtained from Life Technologies (Carlsbad, CA, USA). The cells were grown at 37°C in a humidified atmosphere, with 5% CO_2_. Raw 264.7 cells were stimulated with 100 ng/ml LPS 24 hours. At 0, 6, 9, 15, 20 and 24 hours after LPS stimulation, samples of the conditioned medium of Raw 264.7 cells were collected and the IL12p40 cytokine was quantified by ELISA, following the manufacturing procedures (BioLegend, San Diego, CA, USA).

### Raman & SERS

SERS measurements were carried out with Renishaw inVia Raman microscope. The instrument was equipped with 532 nm excitation laser line and 60x water immersion (NA: 1.0) objective lens. A thermoelectrically cooled charge-coupled device (CCD) was used as detector. All spectra were calibrated with respect to the first-order silicon LO phonon peak at 520 cm^-1^. The spectra were collected in back-scattered geometry at room temperature with a detection range from 561 to 1629 cm^-1^ (spectral resolution of 1.1 cm^-1^). After acquisition, cosmic rays were removed from the spectra with Renishaw WiRE 3.4 software. Processing of the spectra, analysis of the area under curve, smoothing and baseline correction were performed by using the Origin 9.1 software (OriginLab, USA).

### Evaluation of the SERS efficiency

In order to evaluate the SERS efficiency of the Ag nanostructured surfaces, an aqueous solution of 1 μM Rhodamine 6G (R6G) (Sigma-Aldrich, St. Louis, MO, USA) was deposited on Ag islands and Ag flat (40 nm-thick sputtered Ag) substrates for 30 min. After that, substrates were rinsed with water and dried with N_2_. The measurements of SERS were carried out on a 14x12 μm^2^ area (analysed through a 7×8 matrix of spectra with a 2 um step), using a 50x objective, λ = 532 nm laser excitation wave-length, 100 μW nominal laser power and 10 seconds of acquisition time. Experiments were repeated on a total of 24 zones of the sample in order to have statistical consistency.

### SERS on cell conditioned medium

2x10^5^ NIH/3T3 cells or Raw 264.7 cells were seeded in cell culture flasks with 3 ml of complete culture medium. After 1, 2, 3 and 4 days *in vitro* (DIV) for the 3T3 and 6, 9, 15, 20 and 24 hours after LPS stimulation for the Raw 264.7, the conditioned medium was collected, centrifuged at 300g, 3 minutes to avoid dead cells and at 200g, 20 minutes to avoid cell debris. Then, it was transferred into a sterile Petri dish (DxH = 35 mm × 10 mm; surface area 8 cm^2^) containing the Ag nanostructured substrates. As control, 3 ml of culture medium without conditioning were analysed. The SERS measurements were performed with a 60x water immersion objective, using 1 mW laser power and 10 seconds of acquisition time. The spectra were acquired at different points of all substrates. The background was corrected with a multiple point non-linear baseline and spectra were processed with a Savitzky-Golay second derivative-based smoothing (window size of 5 data points with second order polynomial). For each vibrational mode, the same fitting region was used for the deconvolution process, without constraints on peak position and peak width during fitting. The experiment was repeated 4 times and the results of the fitting procedures were averaged in order to have statistical consistency.

### Statistical analysis

Statistical analyses were carried out by means of PCA [43] and cluster analysis [44], considering 500 spectra for each DIV, which include 125 spectra for each replicate. All data are reported as a PC1 *vs* PC2 scatter plot. For each DIV all 4 replicates were considered.

## Results

**Fig 1:**
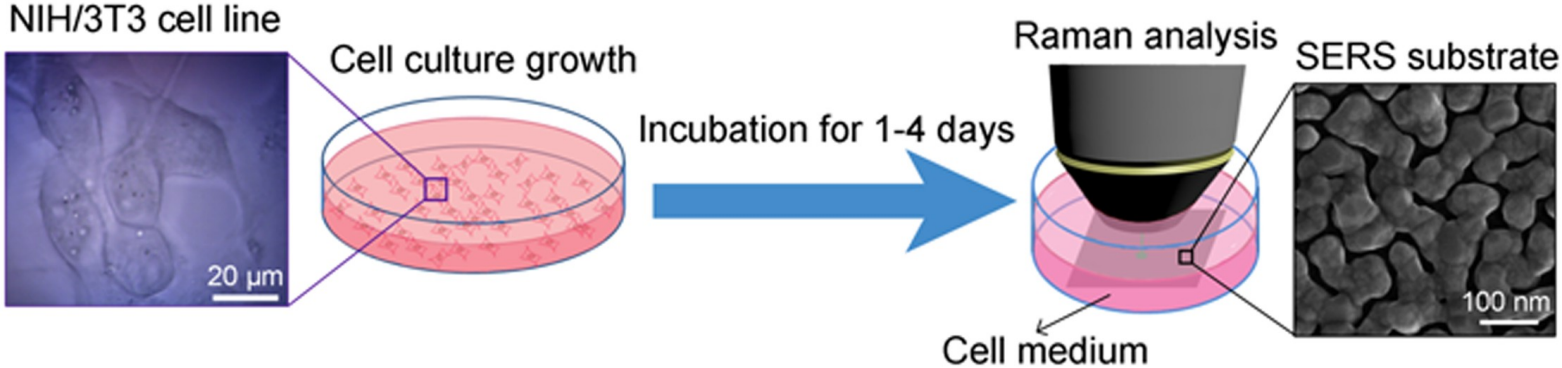
Schematic illustration of the experimental protocol for monitoring of extracellular metabolites. Conditioned medium collected from growing cells is analyzed by Raman spectroscopy using Ag nanostructured substrates. In the insets: an optical image of NIH/3T3 cells (left) and a SEM image of a typical electroless grown Ag nanoisland pattern (right).

In order to test the capability of SERS to analyze the metabolite composition of complex biological samples, such as conditioned culture medium, we developed the following experimental set up, depicted in Fig 1.

2x10^5^ NIH/3T3 cells were seeded in four cell culture dishes with 3 ml of complete culture medium. After 1, 2, 3 and 4 DIVs, the conditioned medium was collected from each dish, centrifuged to avoid dead cells and cell debris, and transferred into a sterile Petri dish with SERS Ag nanostructured substrates for Raman spectroscopy. As control, 3 ml of not conditioned cell medium were analysed as well.

**Fig 2:**
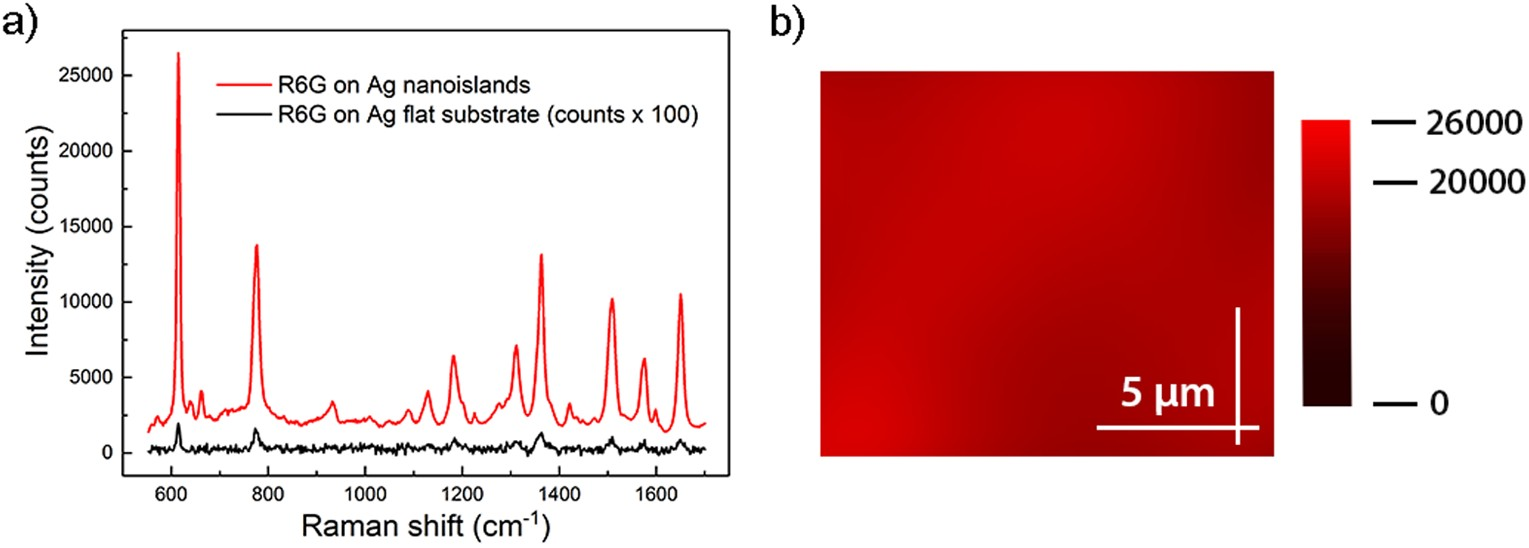
SERS efficiency of the fabricated Ag nanostructured surfaces. (a) SERS spectra of R6G at 1 μM concentration on the Ag nanostructured substrate (red line) and on the Ag flat substrate (black line). (b) 2D SERS mapping centered at the R6G band at 615 cm^-1^. The measurements were done with 532 nm laser line, 100 μW power and 10 second acquisition time.

SERS Ag substrates were prepared by means of electroless deposition of Ag as previously described [41–42]. Detailed description of the fabrication process of the substrates is reported in S1 File. The morphology of Ag nanoislands fabricated following this procedure is represented in the SEM picture of the inset on the right side of Fig 1. The picture shows a few nanometer gap spacing among the Ag nanoislands, known to provide strong local electrical field enhancement [31]. We tested the SERS efficiency of the fabricated Ag nanostructured substrates by using R6G as a probe analyte. As shown in Fig 2A, peaks in the SERS spectrum of R6G at 1 μM concentration obtained on the Ag nanostructured substrate (red line) present higher intensity with respect to the corresponding spectrum obtained on the Ag flat (40 nm sputtered Ag) substrate (black line).

In detail, the R6G peak at 615 cm^-1^ in the spectrum obtained from the Ag nanostructured substrate is ~10^3^ times more intense than the same feature in the spectrum of the Ag flat substrate. Such increase in intensity corresponds to an electric field enhancement factor of about 5–6 times the incoming one, which is a value in agreement with literature data about these kinds of substrates [45–46]. This clearly evidences the high efficiency of the Ag nanostructured surfaces as enhancers of the electromagnetic field in the visible range. Furthermore, in order to verify the uniformity of the Ag nanostructured SERS substrates, 2D spectral maps of the reference band at 615 cm^-1^ covering an area of 14x12 μm^2^ through a 7×8 matrix of spectra with a 2 um step, were collected on 24 different spots of the substrates. Indeed, SERS signal is well known to be highly variable from point to point, thus bringing to misleading interpretation. As show in Fig 2B, the field enhancement efficiency was homogenous throughout the Ag nanostructured substrate. Comparing the SERS intensities on the different spots, variability lower than 5% on the value of field enhancement was found. This evidence supports the Ag nanostructured surface to be an active SERS area with uniformly distributed hot spots.

**Fig 3:**
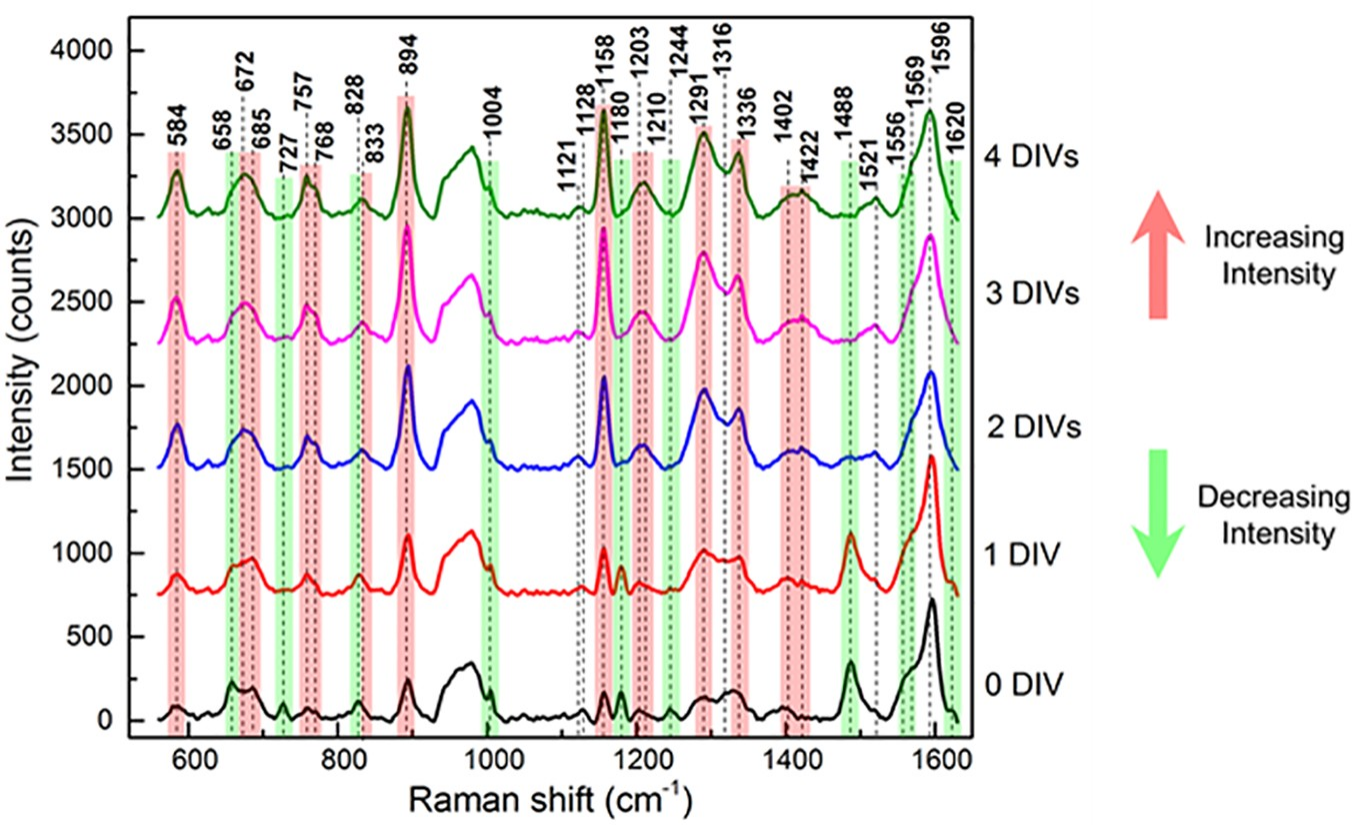
Raman spectra of the 3T3 conditioned medium collected from one to four DIVs. Raman spectra of DMEM culture medium collected at different DIVs of mouse embryonic fibroblasts NIH/3T3 cell line. DIV0 corresponds to complete DMEM without conditioning with cell culture. The measurements were done with 532 nm laser line, 1 mW power and 10 second acquisition time in liquid conditions. The red and green lines indicate the peaks with increasing and decreasing intensities, respectively.

After spectroscopic characterization, the Ag nanostructured substrates were applied for monitoring changes of the metabolites in the cell culture medium over time. Raman spectra of the NIH/3T3 conditioned medium collected from one to four DIVs are shown in Fig 3.

It should be noted that the intensity of almost all of the peaks changed during the incubation time. In particular, the intensity of Raman peaks at 727, 828, 1004, 1180, 1244, 1488 and 1556 cm^-1^ decreased with time (Fig 3, green peaks). On the contrary, the signals at 584, 768, 894, 1158 and 1291 cm^-1^ increased their intensity with time (Fig 3, red peaks). The Raman peaks were attributed to specific metabolites by comparison with the literature data, as reported in Table 1.

**Table 1:** Position and possible corresponding metabolite of the Raman peaks detected in the conditioned culture medium. Raman peaks detected in the cell culture medium and modulated in the intensity during conditioning time are listed. For each of them, the position in the spectrum and the possible corresponding metabolite, in accordance with the literature, are reported.

Peak (cm^-1^)	Possible attribution	References
584	Acetoacetate	[52]
658	Histidine	[48]
685	Proteins	[53]
727	Methionine	[47–48]
757	Cytocrome, Ring breath Tryptophan	[54–55]
768	Fumarate	[52]
828	Tyrosine	[47–48,50–52]
894	Glycine	[47–48,52]
1004	Phenylalanine	[47–48,50–52]
1121	Proteins: stretching CN	[56–57]
1128	Proteins: stretching CN; Carbohydrates: stretching C-O	[55]
1158	Acetoacetate	[52]
1180	Tyrosine	[47–48,50–52]
1203	Phenylalanine	[57]
1210	Nucleic acids: Thymine	[57]
1244	Amide III (β-Sheet)	[49–50]
1291	Fumarate	[52]
1316	Nucleic acids: Guanine; Proteins: C-H; Lipids	[55,58]
1336	Proteins: twisting (CH_2_, CH_3_)	[57]
1402	Deformation CH_3_ asym; Stretching COO^-^	[57]
1422	Nucleic acids: Adenine, Guanine	[56,59]
1488	Histidine	[50]
1521	Nucleic acids: Cytosine	[56]
1556	Tryptophan	[50]
1569	Proteins: Amide II	[56]
1596	Phenylalanine	[56]
1620	Amide I; C = C Tyrosine, Tryptophan, Lipids, stretching (C = C) olefinic	[55–56,60]

The attribution from literature was confirmed by SERS analysis of the pure amino acids, as reported in S2 File. The peaks at 727 and 1244 cm^-1^ can be likely attributed to the FBS components, as confirmed by their detection in the Raman spectrum of the FBS alone, but not in the serum free DMEM, as reported in S3 File. In particular, the 727 cm^-1^ signal is associated to methionine residues [47–48] and that one at 1244 cm^-1^ represents the amide III bound, associated to the β-sheet conformation of 3D-structure of proteins [49–50]. The peaks with decreasing intensity during the conditioning time, at 1004, 1180, 1488 and 1556 cm^-1^, can be attributed to phenylalanine, tyrosine, histidine and tryptophan residues, respectively [47–48,50–52]. Among the peaks with increasing intensity during the conditioning time, the 894 cm^-1^ peak can be ascribed to the ν(CNC) symmetric stretch of the amino group of glycine [47–48,52], the peaks at 584 and 1158 cm^-1^ can be attributed to acetoacetate, while the ones at 768 and 1291 cm^-1^ to fumarate [52]. Other peaks have been attributed to different structural features of analytes, according to references, as reported in Table 1 [53–60].

**Fig 4:**
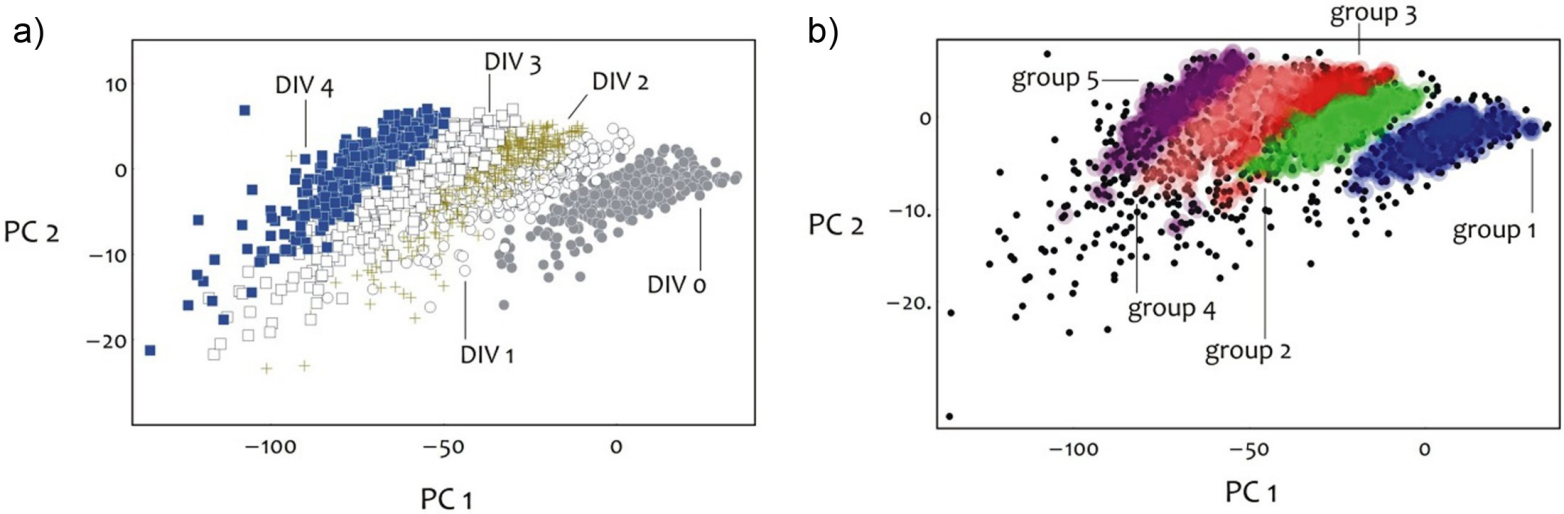
PCA of the Raman spectra from DIV0 to DIV4. PCA of the collected Raman spectra grouped per day (a) and grouped per similarities (b) according to the algorithm developed in [44]. For each DIV, the Raman spectra acquired in all the four replicates are considered.

For the present analysis, hundreds of Raman spectra were acquired and averaged. This massive amount of data may be only partially accessible to the conventional techniques of analysis and a statistical approach, independent of the experience/opinion of the operator, is required. Among the possible mathematical techniques, PCA is one of the most powerful [43]. PCA is a multivariate technique of analysis that can reduce the dimensionality of the original data set, maintaining the variables that most contribute to its variance. Each individual Raman spectrum can be described by a limited number of principal components (PCs), with no loss of information. As consequence, each Raman spectrum of cell medium acquired from DIV0 to DIV4 is plotted as a single dot in function of PC1 and PC2, as shown of Fig 4A.

Notably, in our dataset the Raman spectra acquired during the conditioning time are clearly grouped per DIV, with the spectra collected in each DIV falling into a well-defined region of the plot (Fig 4A). Furthermore, the time distribution of the Raman spectra corresponding to each DIV follows a well-defined trajectory in the PC scatter plot, from the right (DIV0) to the left side (DIV4) of the plot (Fig 4A). This result strongly supports that changes in the Raman spectra during the time of culture are not coincidental but reflect changes in the composition of cell medium.

To verify this hypothesis we exploited a recently developed algorithm that classifies the elements into categories on the basis of their similarity [44]. The result is shown in Fig 4B. Points are colored according to the cluster of the group to which they are assigned. Black points belong to the cluster halos. The fractions of points assigned to the incorrect cluster (that are the assignment errors) across different days are e_(group 1)_ = 0, e_(group 2)_ = 0.035, e_(group 3)_ = 0.023, e_(group 4)_ = 0.0039, e_(group 5)_ = 0.067. The errors are low for each DIV and range from e = 0%, for DIV0, to e = 6.7%, for DIV 4. In the S4 File, the cluster analysis is described in details. The bar charts in S5 Fig describe the intra cluster variance as a function of cluster number or DIV, while S6 Fig represents the PC1 vs PC2 scatter plot for different replicates across all the considered time points. One may observe that the point kins to different culture groups overlap over wide regions of the diagram (S6 Fig). This indicates that the replicate-to-replicate variation is low. The very low mismatch (e≤6.7%) between the original data points grouped per DIV and their unbiased classification by similarities confirms that our hypothesis can be accepted with a high level of confidence, suggesting that each region in the PC plot represents a metabolic status or “age” (DIV).

**Fig 5:**
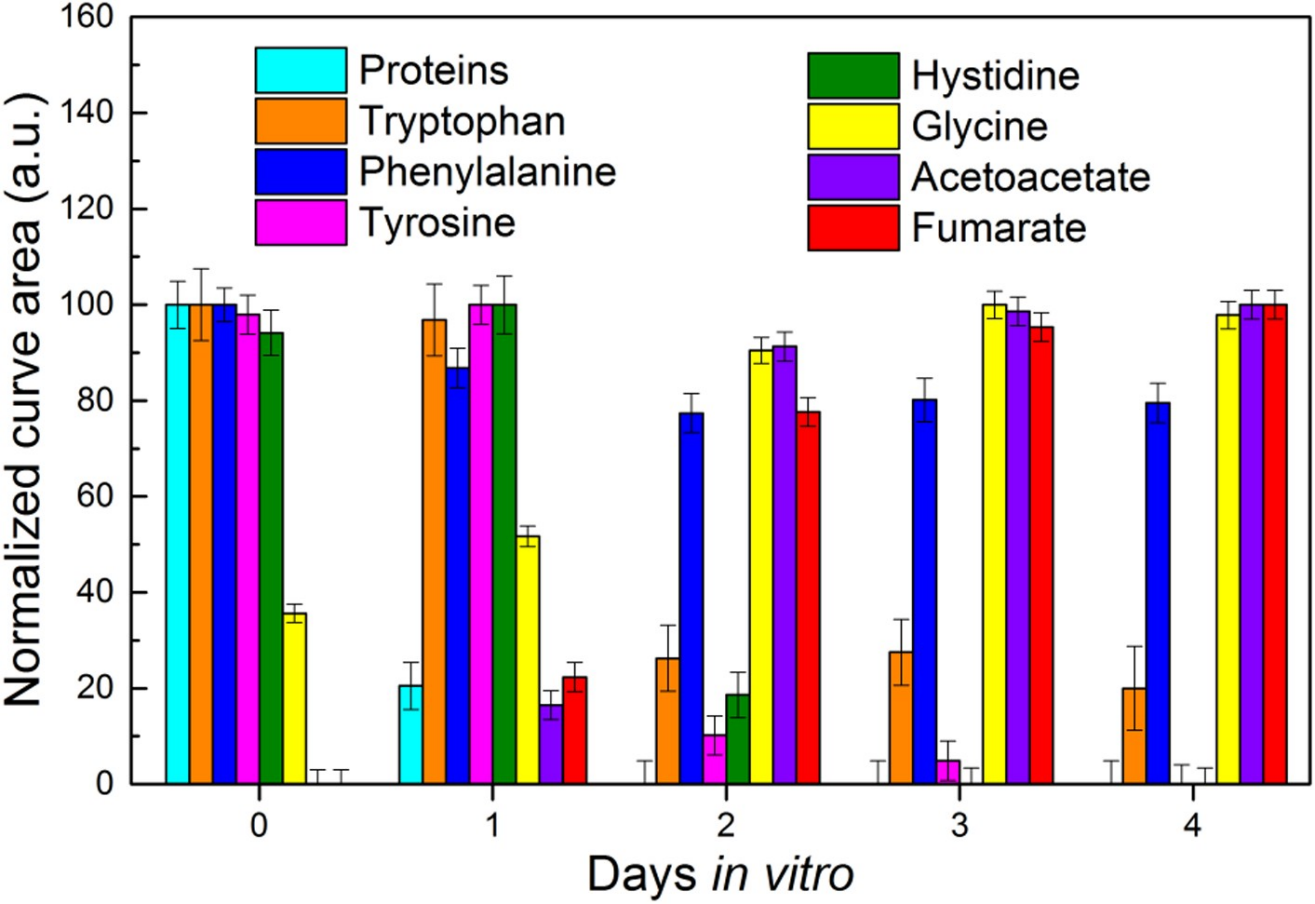
Analysis of the intensity for multiple Raman peaks of the cell conditioned medium. The normalized integral curve areas of peaks corresponding to different analytes present in cell culture medium are reported from DIV0 to DIV4. Each color corresponds to a different analyte.

Finally, we compared the intensity changes for peaks referred to different analytes, namely the FBS proteins (represented by the methionine residues), L-tryptophan, L-phenylalanine, L-tyrosine, L-histidine, glycine, acetoacetate and fumarate (Fig 5 and S5 File).

We extracted the values of the area under the peak at 727, 1556, 1004, 1180, 1488, 894, 584 and 768 cm^-1^, respectively. For each peak, we averaged the values from the four sample replicates and we normalized to the lowest value. Interestingly, the signals referred to the acetoacetate and fumarate metabolites, the two end products of L-tyrosine degradation [61–62], increased concurrently to the decrease of L-tyrosine levels (Fig 5). At DIV2 the normalized values of the L-tyrosine peak area rapidly lowered (Fig 5, pink column), while the values of acetoacetate and fumarate sharply raised up (Fig 5, purple and red columns respectively). These inverse trends likely describe the consumption of L-tyrosine during the conditioning time, with the corresponding production of acetoacetate and fumarate, supporting the reliability of the method.

Although it is still a challenge to use SERS as a quantitative technique, it is possible to perform calibration tests to determine the metabolite concentrations [63]. For instance, as reported in in S6 File, analyzing multiple solutions with increasing concentrations of L-Tyrosine, from 0.5 to 10 μM, the detected SERS signals of L-tyrosine were linearly increasing in intensity with the concentration of the analyte. As shown, the results are quantitative in the 0.5–10 μM range of concentrations and provide a sensitive method of detecting L-tyrosine in solution.

**Fig 6:**
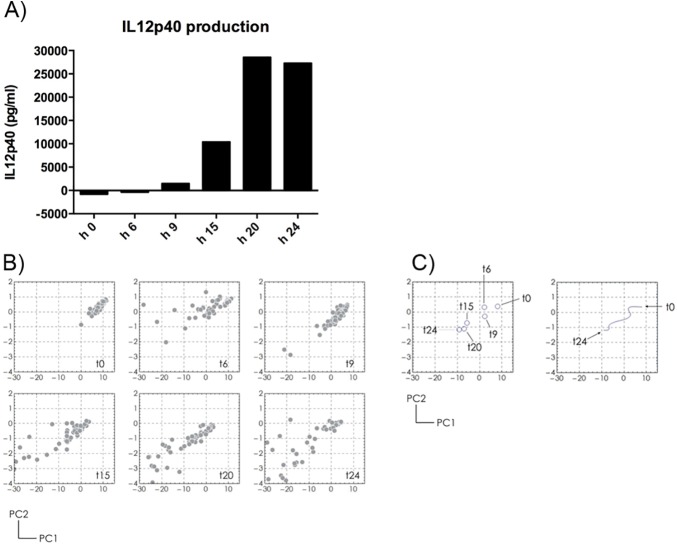
Analysis of the Raw 264.7 conditioned medium after LPS stimulation. Raw 264.7 cells are stimulated with LPS for 24 hours. At 0, 6, 9, 15, 20, 24 hours, A) IL12p40 production was quantified by ELISA and B) Raman spectra of conditioned medium were collected. By PCA analysis, the spectra collected at each time point clusterize. C) The cluster centers for each time point are reported (left panel) as well as the trajectory described (right panel).

In addition to the SERS characterization of cell culture conditions, we investigated whether our strategy could be applied for an indirect monitoring of the functional state of cells in culture over time. To test this hypothesis we cultured the Raw 264.7, a macrophage cell line, in presence of the LPS, a pro-inflammatory stimulus that induces a functional switch of the macrophages from a “quiescent” state to a “classically activated” pro-inflammatory state. The polarization toward an inflammatory state has been monitored through the quantification by ELISA of the IL12p40, released by the cells in the conditioned medium. As shown in Fig 6A, the IL12p40 production by Raw 264.7 increases during 24 hours of LPS stimulation, confirming the functional switch to the inflammatory state.

At the same time, the Raman spectra of the conditioned medium collected at 6, 9, 15, 20 and 24 hours of LPS stimulation, analyzed with PCA analysis, clusterize in different regions of the PC plot (Fig 6B), describing a well-defined trajectory (Fig 6C, left and right graphs), reflecting the functional change of the cells.

As an alternative to the experimental approach described above, we also tested whether the nanostructured substrates could be placed in the culture dishes together with the cells, instead of the conditioned medium only. 2x10^5^ NIH/3T3 cells or Raw 264.7 cells were seeded in cell culture dishes on the Ag nanostructured substrates and cultured two days. Then, the compatibility of Ag nanostructured surfaces was tested through the LIVE/DEAD Viability/Cytotoxicity cell assay. As reported in S7 File, after 2 DIVs the Ag SERS substrates did not evidence cytotoxic effects on NIH/3T3 or Raw 264.7 cells, evaluated through the number of dead cells (S9 Fig), and the morphology of cells grown on the substrates, for the NIH/3T3 cells (S10 Fig). These data suggest that the Ag nanostructured substrates could be placed directly into the cell culture system for monitoring the metabolite composition of the conditioned medium.

## Conclusions

In this study we proposed a straightforward approach for time resolved monitoring of metabolite dynamics in cell cultures, based on the SERS spectroscopic technique, for monitoring cell culture conditions and functional changes of cells. In comparison to the current available techniques, such as MS and NMR, this method provides several advantages. Indeed, it is relatively easy, label-free and non-destructive for the biological samples. For these reasons, differently from the MS and NMR, it can be particularly convenient for long term monitoring of analytes in biological samples available only in limited amount.

As reported in literature, even though SERS-based techniques have been widely explored for the analysis of biological samples for different purposes, these studies are limited to the analysis of few biomolecules only. At the best of our knowledge, this work represents one of the first attempts to obtain a complete mapping of the cell culture medium composition over time through SERS. Indeed, several analytes in the cell conditioned medium are simultaneously monitored over time, such as amino acids (i.e. phenylalanine, tyrosine, histidine and tryptophan), metabolites (i.e. acetoacetate and fumarate) and serum proteins. Moreover, our substrates are fabricated through a one step, scalable and cheap procedure, allowing for their potential integration in commercial microfluidic devices.

In this work, the conditioned extracellular media collected from growing cells are analysed by Raman spectroscopy onto Ag nanostructured substrates. Through dedicated calibration tests, we show that our nanostructured surfaces represent a powerful and reliable biosensor with two important features. First, our Ag-nanostructured substrates present uniformly distributed SERS activity throughout their surfaces Second, our strategy can be utilized within a range of almost two order of concentrations, as quantitative SERS-based method, in order to determine metabolite concentrations.

On the basis of these features, we could successfully applied our Ag-nanostructured substrates to monitor by SERS the changes in the composition of cell conditioned medium over time. As verified by reference solutions of pure amino acids, changes in the Raman spectra, such as increases or decreases of Raman peak intensities, are consistent with the different duration of metabolic reactions related to the cell growth, from one to four DIVs, for the NIH/3T3 cells. The Raman peaks at 727, 828, 1004, 1180, 1244, 1488 and 1556 cm^-1^ with decreasing intensity over time, likely indicate the cell medium components exhausted during the conditioning time. On the contrary, the signals at 584, 768, 894, 1158 and 1291 cm^-1^ with increasing intensity, are likely related to different metabolites accumulated during the cell growth. In details, we showed the inverse trends over time for the peaks of L-tyrosine and its end products of degradation, acetoacetate and fumarate, describing the consumption of L-tyrosine and the production of its degradation products. In addition, as verified by the PCA of the acquired data, the time evolution of Raman spectra corresponds to a well-defined trajectory in the PC plot, as highlighted by the clustering algorithm based on similarities. This trajectory reflects the changes over time in the composition of the cell medium, supporting the idea that each region of the PC plot can represent a metabolic status of the cells. The obtained results suggest how engineered SERS surfaces can be used as a valid tool for multi-analyte detection and represent as well a versatile biosensor able to probe the “age” of cell systems. Therefore, it is conceivable to potentially use this system to distinguish pathological *versus* physiological biosamples, as well as the activation/differentiation states of a cell type, i.e. quiescent or activated lymphocytes [7]. Unlike fluorescence-based methods, or other conventional biochemical approaches, this SERS method could be expanded to measure numerous bio-components simultaneously and incorporated into an in-line, time resolved monitoring strategy.

We finally investigated whether our strategy could be applied for indirect monitoring of functional state of cells in culture over time. The trajectory described by the clustered Raman spectra reflected the changes of the Raw 264.7 conditioned medium induced by the LPS, corresponding to a functional change of cells from the quiescent to the activated state.

As shown with the Raw 264.7 cells stimulated with LPS, the Raman spectra of the conditioned medium collected at different time points over 24 hours, clusterized in different regions of the PC plot, describing a well-defined trajectory. This distribution reflected the simultaneous functional change of cells, polarized toward a pro-inflammatory profile induced by the LPS, demonstrated by the IL12p40 quantification.

In conclusion, the overall data presented in this study support our strategy as a feasible, powerful and reliable SERS-based method for a qualitative and partially quantitative monitoring of the changes in the cell culture conditioned medium. Moreover, through the development of customized protocols, the methodology could be applied also for an indirect description of the functional status of cells over time.

## Supporting information

S1 File Fabrication process. (DOCX)

S2 File Identification of the pure amino acid peaks in the Raman spectra of the NIH/3T3 conditioned medium. (DOCX)

S3 File FBS and DMEM Raman spectra. (DOCX)

S4 File Statistical analysis. (DOCX)

S5 File Additional Raman peak variations. (DOCX)

S6 File Calibration test. (DOCX)

S7 File Biocompatibility. (DOCX)

S1 Fig Fabrication details. Scheme of the electroless deposition of Ag nanoparticles aggregates in water solution of AgNO_3_ and HF. (A) Redox reaction between Ag^+^ and Si: Ag^+^ ions in the vicinity with the silicon surface capture electrons from the valence band of Si. (B) Ag^+^ ions are reduced and deposited as metals while the silicon surface is oxidized into SiO_2_. (C) The redox reaction involves hydrofluoric acid, which induces the etching of SiO_2_ and the dissolution of SiF^2−^. (D) The Ag nuclei attract electrons from bulk silicon, become as a catalytic surface for the reduction of further Ag^+^ ions. (E) SEM images of a typical electroless grown Ag nanoislands pattern, the form and size of nanoislands is a function of the deposition time.
(TIF)

S2 Fig Identification of the pure amino acid peaks in the Raman spectra of the NIH/3T3 conditioned medium. Raman spectra of the pure amino acids Tyrosine, Histidine, Tryptophane, Glycine, Phenylalanine are compared to the Raman spectra of the complete medium at DIV 0 and DIV 4. The characteristic peaks of each amino acid are highlighted in the medium spectra with different colors.
(TIF)

S3 Fig SERS analysis of FBS and DMEM. SERS spectrum of the separate cell medium components on the nanoislands Ag substrate: (A) Fetal bovine serum (FBS); (B) Dulbecco's modified Eagle's medium (DMEM) without red phenol.
(TIF)

S4 Fig Cluster center determination. The diagram showing the cluster centers determination: cluster centers are the points in the set which have higher density respect to their neighbors and a relatively large distance from points with higher densities.
(TIF)

S5 Fig Cluster variability. The variability within clusters presented in Fig 4B of the main text.
(TIF)

S6 Fig Scatter plot. The PC1 vs PC2 scatter plot for different replicates across all the considered time points.
(TIF)

S7 Fig Raman peak variation. The rates of normalized integral curve areas of the peaks resolved in the spectral ranges: 640–716 cm^-1^ (A), 1190–1240 cm^-1^ (B), 1460–1629 cm^-1^ (C), 737–790 cm-1, 1250–1363 cm-1 and 1368–1460 cm-1 (D).
(TIF)

S8 Fig Calibration measurements for L-Tyrosine detection. (A) SERS spectra of L-Tyrosine at the concentrations 0.1–10 μM. (B) L-Tyrosine band at 1162 cm-1. (C) Linear fitting of 1162 cm-1 peak curve area versus concentration. The measurements were done with 532 nm laser line, 100 μW power and 10 s acquisition time in liquid conditions.
(TIF)

S9 Fig Live/dead assay. Live/dead staining of NIH/3T3 (A) and Raw 264.7 (B) cells grown on Ag nanostructured substrates at 2 DIVs. (a) Black columns indicate the percentage of live cells, while the white columns represent the percentage of dead cells. ****p ≤ 0.0001 and ***p ≤ 0.0002. (b) The viability of NIH/3T3 and Raw 264.7 cells is evidenced by green live cells, in comparison to the red dead cells, on glass coverslips and Ag nanostructured substrates. Scale bar: 100 μm.
(TIF)

S10 Fig SEM of cells. SEM images of NIH/3T3 cells fixed at DIVs 2 on Ag island films. The cells are well-spread and show a flat morphology with well-visible filopodia. Scale bar: 20 μm.
(TIF)
