# Supplementary material for: Time resolved and label free monitoring of extracellular metabolites by surface enhanced Raman spectroscopy
Source: PLoS One. 2017 Apr 18;12(4):e0175581. doi: 10.1371/journal.pone.0175581 (PMC5395151; doi:10.1371/journal.pone.0175581)
Supplement: S1 File — (DOCX) [file pone.0175581.s001.docx]

Supporting Information 1

Time resolved and label free monitoring of extracellular metabolites by surface-enhanced Raman spectroscopy

Victoria Shalabaeva^1^, Laura Lovato^1*^, Rosanna La Rocca^1^, Gabriele C. Messina^1^, Michele Dipalo^1^, Ermanno Miele^1^, Michela Perrone^1^, Francesco Gentile^2^, Francesco De Angelis^1*^

^1^ Plasmon Nanotechnologies, Istituto Italiano di Tecnologia, Genoa, Italy.

^2^ Department of Electrical Engineering and Information Technologies (DIETI), University Federico II of Naples, Naples, Italy.

^*^Corresponding authors:

E-mail:francesco.deangelis@iit.it (FDA); laura.lovato@iit.it (LL)

**Fabrication process**

Silver films were prepared by means of electroless deposition of Ag.^1,2^ This process consists in the immersion of a silicon substrate with native oxide in a water solution containing fluoride acid and Ag nitrate. The scheme of the electroless chemical reaction is briefly summarized in S1 Fig. Here, the reducing agent is the substrate itself, silicon, that oxidizes and reduces Ag ions to the metallic forms, as described by the following chemical reaction:

$4{Ag}^{+}+Si+6HF \to4{Ag}^{0}+ H_{2}SiF_{6}+4H^{+}$ (1)

which can be also divided by following reactions, that are the Si oxidation, as the anode:

$Si+2H_{2}O \to SiO_{2}+4H^{+}+4e^{-}$ (2)

silicon dissolution:

$SiO_{2}+6HF \to2H^{+}+SiF_{6}^{2-}+2H_{2}O$ (3)

and the Ag reduction, at the cathode:

${Ag}^{+}+ e^{-} \to{Ag}^{0}$ (4)

The HF reacts with the SiO_2_ and initiates a serie of steps that eventually lead to the transfer of electrons from the silicon to the solution. The Ag^+^ ions from the dissociated salt, diffused to the surface, are reduced by electrons to Ag^0^ which is then adsorbed on the surface through van der Waals interactions. The growing metallic Ag structures act as an electrode charged by the electrons in the silicon and further contribute to the reduction of Ag ions from the solution.

The overall deposition was performed with two minutes of incubation of Si samples in the AgNO_3_ 1 mM and HF 0,15 M solution at 50°C with vigorous agitation at 100 rpm. These parameters allowed an optimal control of the deposition and have been determined via several preliminary experiments. To gain information about the intermediate stages of the growth, the deposition process can be stopped before the formation of the full structure. SEM images reported in S1E Fig reveal that the Ag nano-islands formed on the substrate were separated by few nanometer gap spacing, a morphology desirable for local electrical field enhancement and SERS, and proof of the long-range uniformity as well as of a good reproducibility of the surfaces.

**S1 Fig**. **Fabrication details.** Scheme of the electroless deposition of Ag nanoparticles aggregates in water solution of AgNO_3_ and HF. (A) Redox reaction between Ag^+^ and Si: Ag^+^ ions in the vicinity with the silicon surface capture electrons from the valence band of Si. (B) Ag^+^ ions are reduced and deposited as metals while the silicon surface is oxidized into SiO_2_. (C) The redox reaction involves hydrofluoric acid, which induces the etching of SiO_2_ and the dissolution of SiF^2−^. (D) The Ag nuclei attract electrons from bulk silicon, become as a catalytic surface for the reduction of further Ag^+^ ions. (E) SEM images of a typical electroless grown Ag nanoislands pattern, the form and size of nanoislands is a function of the deposition time.

**References**

1. F. De Angelis, C. Liberale, M. L. Coluccio, G. Cojoc and E. Di Fabrizio, *Nanoscale*, 2011, **3,** 2689–2696.

2. M. L. Coluccio, F. Gentile, M. Francardi, G. Perozziello, N. Malara, P. Candeloro and E. Di Fabrizio, *Sensors (Basel)*, 2014, **14**, 6056–6083.
