# Supplementary material for: Time resolved and label free monitoring of extracellular metabolites by surface enhanced Raman spectroscopy
Source: PLoS One. 2017 Apr 18;12(4):e0175581. doi: 10.1371/journal.pone.0175581 (PMC5395151; doi:10.1371/journal.pone.0175581)
Supplement: S2 File — (DOCX) [file pone.0175581.s002.docx]

Supporting Information 2

Time resolved and label free monitoring of extracellular metabolites by surface-enhanced Raman spectroscopy

Victoria Shalabaeva^1^, Laura Lovato^1*^, Rosanna La Rocca^1^, Gabriele C. Messina^1^, Michele Dipalo^1^, Ermanno Miele^1^, Michela Perrone^1^, Francesco Gentile^2^, Francesco De Angelis^1*^

^1^ Plasmon Nanotechnologies, Istituto Italiano di Tecnologia, Genoa, Italy.

^2^ Department of Electrical Engineering and Information Technologies (DIETI), University Federico II of Naples, Naples, Italy.

^*^Corresponding authors:

E-mail:francesco.deangelis@iit.it (FDA); laura.lovato@iit.it (LL)

**Identification of the pure amino acid peaks in the Raman spectra of the NIH/3T3 conditioned medium.**

**S2 Fig. Identification of the pure aminoacid peaks in the Raman spectra of the NIH/3T3 conditioned medium.** Raman spectra of the pure amino acids Tyrosine, Histidine, Tryptophane, Glycine, Phenylalanine are compared to the Raman spectra of the complete medium at DIV 0 and DIV 4. The characteristic peaks of each amino acid are highlighted in the medium spectra with different colors.
