# Supplementary material for: Time resolved and label free monitoring of extracellular metabolites by surface enhanced Raman spectroscopy
Source: PLoS One. 2017 Apr 18;12(4):e0175581. doi: 10.1371/journal.pone.0175581 (PMC5395151; doi:10.1371/journal.pone.0175581)
Supplement: S3 File — (DOCX) [file pone.0175581.s003.docx]

Supporting Information 3

Time resolved and label free monitoring of extracellular metabolites by surface-enhanced Raman spectroscopy

Victoria Shalabaeva^1^, Laura Lovato^1*^, Rosanna La Rocca^1^, Gabriele C. Messina^1^, Michele Dipalo^1^, Ermanno Miele^1^, Michela Perrone^1^, Francesco Gentile^2^, Francesco De Angelis^1*^

^1^ Plasmon Nanotechnologies, Istituto Italiano di Tecnologia, Genoa, Italy.

^2^ Department of Electrical Engineering and Information Technologies (DIETI), University Federico II of Naples, Naples, Italy.

^*^Corresponding authors:

E-mail:francesco.deangelis@iit.it (FDA); laura.lovato@iit.it (LL)

**FBS and DMEM Raman spectra**

**S3 Fig**. **SERS analysis of FBS and DMEM.** SERS spectrum of distinct cell medium components on the nanoislands Ag substrate: (A) Fetal bovine serum (FBS); (B) DMEM without red phenol.
