# Supplementary material for: Time resolved and label free monitoring of extracellular metabolites by surface enhanced Raman spectroscopy
Source: PLoS One. 2017 Apr 18;12(4):e0175581. doi: 10.1371/journal.pone.0175581 (PMC5395151; doi:10.1371/journal.pone.0175581)
Supplement: S4 File — (DOCX) [file pone.0175581.s004.docx]

Supporting Information 4

Time resolved and label free monitoring of extracellular metabolites by surface-enhanced Raman spectroscopy

Victoria Shalabaeva^1^, Laura Lovato^1*^, Rosanna La Rocca^1^, Gabriele C. Messina^1^, Michele Dipalo^1^, Ermanno Miele^1^, Michela Perrone^1^, Francesco Gentile^2^, Francesco De Angelis^1*^

^1^ Plasmon Nanotechnologies, Istituto Italiano di Tecnologia, Genoa, Italy.

^2^ Department of Electrical Engineering and Information Technologies (DIETI), University Federico II of Naples, Naples, Italy.

^*^Corresponding authors:

E-mail:francesco.deangelis@iit.it (FDA); laura.lovato@iit.it (LL)

**Statistical analysis**

**PCA algorithm**

Let us assume that the data are arranged in a matrix $\boldsymbol{X}_{p\times n}$, where the rows $(p)$ are the frequency points over which each spectrum is represented and the columns $(n)$ are the Raman spectra acquired over the entire time course of the experiment, from DIV $0$ to DIV $4$. Thus $n$ comprehends five groups of $n_{1}$ to $n_{5}$ independent observed spectra per DIV ($n=\sum_{i=1}^{5} n_{i}$). If the groups belong to different populations, the PCA analysis would differentiate among them. We represent $\boldsymbol{X}_{o}$ as

| $\boldsymbol{X}_{o}=\left( \begin{matrix} x_{11} & x_{12} & . & x_{1n} \\ x_{21} & x_{22} & . & x_{2n} \\ . & . & . & . \\ x_{p1} & x_{p2} & . & x_{pn} \end{matrix} \right).$ | (5) |
| --- | --- |

First, each element is centered on the average value of that element calculated over all the spectra:

| $X\left( i,j \right)=X_{o}\left( i,j \right)-\frac{1}{n}\sum_{j=1}^{n} X\left( i,j \right)$. | (6) |
| --- | --- |

Then, we derive a bivariate measure of dispersion of $\boldsymbol{X}_{p\times n}$ calculated as its covariance matrix:

| $\boldsymbol{C}_{X}\left( i,j \right)=\frac{1}{n-1}\sum_{k=1}^{n} \left( X\left( i,k \right)-\left\langle X\left( i,k \right) \right\rangle\right)\left( X\left( j,k \right)-\left\langle X\left( j,k \right) \right\rangle\right)=\frac{1}{n-1}\left( \boldsymbol{X}\cdot\boldsymbol{X}^{+} \right),$ | (7) |
| --- | --- |

where $\left\langle\right\rangle$ is the average operator, while the superscript on $\boldsymbol{X}$ means that we conjugate transpose $\boldsymbol{X}$. To find the maximum variance associated to $\boldsymbol{C}_{X}$, we calculate the eigenvalues $\lambda$ and eigenvectors $\boldsymbol{V}$ of $\boldsymbol{C}_{X}$ using the well-known relationships:

| $\left\vert\boldsymbol{C}_{X}-\lambda_{r}\boldsymbol{I} \right\vert=0$,  $\lambda_{r}\boldsymbol{V}_{r}=\boldsymbol{C}_{X}\cdot\boldsymbol{V}_{r}, r=1,\ldots, p$. | (8) |
| --- | --- |

And, rearranging in matrix form:

| $\boldsymbol{D}=\left( \begin{matrix} \lambda_{1} & 0 & . & 0 \\ 0 & \lambda_{2} & . & 0 \\ . & . & . & . \\ 0 & 0 & . & \lambda_{p} \end{matrix} \right), \boldsymbol{V}=\left( V_{1},V_{2},\ldots, V_{p} \right)$. | (9) |
| --- | --- |

The covariance matrix $\boldsymbol{C}_{X}$, the eigenvalues matrix $\boldsymbol{D}$ and the eigenvectors matrix **V** are combined in a single formula:

| $\boldsymbol{V}^{\boldsymbol{-1}} \boldsymbol{C}_{X}\mathbf{V=D}$. | (10) |
| --- | --- |

The eigenvalues are sorted in order of decreasing value $\lambda_{i}>\lambda_{i+1}$. Under these conditions, the eigenvalues $\lambda_{i}$ account for the maximum variance of the spectra: they describe the energy content of the original dataset represented within the new basis defined by the eigenvectors $V_{i}$. The cumulative energy can be normalized to unity:

| $f\left( l \right)={\sum_{i=1}^{l} \lambda_{i}}/{\sum_{i=1}^{p} \lambda_{p}, l<p}$ | (11) |
| --- | --- |

Even if $p$ is big, like as $p\mathcal{=o}\left( 1000 \right)$, only the first few values of $\lambda$ account for most of the energy content of the spectra. In what follows, we disregard all the eigenvalues $\lambda_{j}:\left( j>q \right)\bigwedge\left( f\left( q \right)=t \right)$, where a typical value of the threshold $t$ is $t=0.9$. In this way, we maintain the eigenvalues that describe the $90\%$ of the energy of the spectra. In doing so, we reduce the problem from $p$ to $q$ degrees of freedom, with generally $q\ll p$.

| $\boldsymbol{D}_{p\times p}\boldsymbol{\to}\boldsymbol{D}_{q\times q}^{o}\boldsymbol{,}\boldsymbol{V}_{p\times p}\boldsymbol{\to}\boldsymbol{V}_{p\times q}^{o}$**,** | (12) |
| --- | --- |

$\boldsymbol{V}_{p\times q}^{o}$ represents the new basis in which the measured Raman spectra exhibit the maximum components of variance. The original data are then normalized with respect to their variance, and projected into the new basis:

| $\boldsymbol{X}_{p\times n}^{\boldsymbol{(}2\boldsymbol{)}}\boldsymbol{=}{\boldsymbol{X}_{p\times n}}/{\boldsymbol{S}_{p\times n}}$**;** $\boldsymbol{Y}_{q\times n}\boldsymbol{=}{\boldsymbol{V}_{q\times p}^{o}}^{\boldsymbol{+}}\boldsymbol{\cdot}\boldsymbol{X}_{p\times n}^{\boldsymbol{(}2\boldsymbol{)}}\boldsymbol{=}KLT\left( \boldsymbol{X} \right).$ | (13) |
| --- | --- |

Where $S$ is the empirical standard deviation matrix created from the square root of each element along the main diagonal of the diagonalized covariance matrix $C_{X}$

| $\boldsymbol{S}_{p\times n}\boldsymbol{=}\left( \begin{matrix} \sqrt{\boldsymbol{C}_{X}\left( 1,1 \right)} \\ \begin{matrix} \sqrt{\boldsymbol{C}_{X}\left( 2,2 \right)} \\ \vdots\end{matrix} \\ \boldsymbol{C}_{X}\left( p,p \right) \end{matrix} \right)_{p\times1}\boldsymbol{\cdot}\left( \begin{matrix} 1 & 1 & 1 \end{matrix} \begin{matrix} \cdots& 1 \end{matrix} \right)_{1\times n}$ | (14) |
| --- | --- |

PCA thus is an orthogonal linear transformation that transforms the original data into a new basis in a way that the new coordinates are uncorrelated: PCA is also called the Karhunen-Loève transform ($KLT$), $Y=KLT\left( \boldsymbol{X} \right)$. $\boldsymbol{Y}$ comprises $n$ columns of length $q$: each column is a Raman spectrum described through $q$ *Principal Components*.^1^ If the first of these components are reported in a scatter plot, we can appreciate differences among groups. Notice that subtraction from the average as in Equation (6) and normalization of data with respect to the covariance matrix as in Equations (13-14) (where elements along the main diagonal account for the variations over all different measurements at a fixed dimension) compensate Raman intensity fluctuations due to spatial inhomogeneity of the SERS substrate. Measurements performed over a large number of repetitions n>600 allow random spatial errors to cancel each other out, and their sum approaches zero.

**Clustering analysis**

We partitioned elements into groups using a recently developed density based clustering algorithm.^2^ The algorithm classifies elements into categories on the basis of their similarity. Cluster centers are determined as those points in the set which have higher density respect to their neighbors and by a relatively large distance from points with higher densities. To do so, per each point $o$ in the set: (i) we determine its density $\rho\left( o \right)$ as the number of points that are closer than a cut off distance $\delta_{co}$ to $o$; (ii) we find the subset $s \epsilon S$ of points in the dataset with densities $\rho\left( s \right)>\rho\left( o \right)$; (iii) we find the point $a \epsilon S$ with minimum distance to $o$, this distance is $\delta_{min/\rho}\left( o \right)$: the minimum distance of $o$ from points with higher densities than $o$. After operations from (i) to (iii), we derive a diagram where the density $\rho$ is reported against $\delta_{min/\rho}$ per each element in the data set. Points in the set with higher density than their neighbors and a relatively large distance from points with higher densities emerge as singularities in the diagram, an example of which is reported in S3 Fig.

S4 Fig. Cluster center determination. The diagram showing the cluster centers determination: cluster centers are the points in the set which have higher density respect to their neighbors and a relatively large distance from points with higher densities.

These points are the cluster centers. Each point in the set is attributed to different clusters on the basis of a minimum distance criterion: a point $b$ is attributed to a cluster $G_{i}$ if the minimum distance of $b$ to $G_{i}$ is the smaller among all the minimum distances calculated with the remaining clusters. Thus clusters are constructed per accumulation. The cluster centers represent the seeds of the clusters. In Fig 4b in the main article, points are colored according to the cluster of the group to which they are assigned. Black points belong to the cluster halos. The bar charts in S4 Fig describe the intra cluster variance (that is, the variability within groups) as a function of cluster number or DIV.

S5 Fig. Cluster variability. The variability within clusters presented in Fig 4b of the main text.

It is noticeable that the variance changes with the number of DIVs and this may reflect an increasing metabolic activity up to day 3. Instead, after day 3 a contraction in the intra cluster variability is observed that can be ascribed to a decline of the rate of the overall metabolic reaction.

The PC1 vs PC2 scatter plot for different replicates across all the considered time points is presented in S6 Fig.

S6 Fig. Scatter plot. The PC1 vs PC2 scatter plot for different duplicates across all the considered time points.

One may observe that the points, kin to different culture groups, overlap over wide regions of the diagram. This indicates that culture-to-culture, or duplicate-to-duplicate, variation is low.

**References**

1. G. Das, F. Gentile, M. L. Coluccio, A. M. Perri, A. Nicastri, F. Mecarini, G. Cojoc, P. Candeloro, C. Liberale, F. De Angelis and E. Di Fabrizio, *J. Mol. Struct.*, 2011, **993**, 500–505.

2. A. Rodriguez and A. Laio, *Science*, 2014, **344**, 1492–1496.
