# Supplementary material for: Time resolved and label free monitoring of extracellular metabolites by surface enhanced Raman spectroscopy
Source: PLoS One. 2017 Apr 18;12(4):e0175581. doi: 10.1371/journal.pone.0175581 (PMC5395151; doi:10.1371/journal.pone.0175581)
Supplement: S5 File — (DOCX) [file pone.0175581.s005.docx]

Supporting Information 5

Time resolved and label free monitoring of extracellular metabolites by surface-enhanced Raman spectroscopy

Victoria Shalabaeva^1^, Laura Lovato^1*^, Rosanna La Rocca^1^, Gabriele C. Messina^1^, Michele Dipalo^1^, Ermanno Miele^1^, Michela Perrone^1^, Francesco Gentile^2^, Francesco De Angelis^1*^

^1^ Plasmon Nanotechnologies, Istituto Italiano di Tecnologia, Genoa, Italy.

^2^ Department of Electrical Engineering and Information Technologies (DIETI), University Federico II of Naples, Naples, Italy.

^*^Corresponding authors:

E-mail:francesco.deangelis@iit.it (FDA); laura.lovato@iit.it (LL)

**Additional Raman peak variations**

**S7 Fig. Raman peak variation.** The rates of normalized integral curve areas of the peaks resolved in the spectral ranges: 640-716 cm^-1^ (A), 1190-1240 cm^-1^ (B), 1460-1629 cm^-1^ (C), 737-790 cm^-1^, 1250-1363 cm^-1^ and 1368-1460 cm^-1^ (D).
