# Supplementary material for: Time resolved and label free monitoring of extracellular metabolites by surface enhanced Raman spectroscopy
Source: PLoS One. 2017 Apr 18;12(4):e0175581. doi: 10.1371/journal.pone.0175581 (PMC5395151; doi:10.1371/journal.pone.0175581)
Supplement: S6 File — (DOCX) [file pone.0175581.s006.docx]

Supporting Information 6

Time resolved and label free monitoring of extracellular metabolites by surface-enhanced Raman spectroscopy

Victoria Shalabaeva^1^, Laura Lovato^1*^, Rosanna La Rocca^1^, Gabriele C. Messina^1^, Michele Dipalo^1^, Ermanno Miele^1^, Michela Perrone^1^, Francesco Gentile^2^, Francesco De Angelis^1*^

^1^ Plasmon Nanotechnologies, Istituto Italiano di Tecnologia, Genoa, Italy.

^2^ Department of Electrical Engineering and Information Technologies (DIETI), University Federico II of Naples, Naples, Italy.

^*^Corresponding authors:

E-mail:francesco.deangelis@iit.it (FDA); laura.lovato@iit.it (LL)

**Calibration test**

The calibration measurements were performed in order to find the relationship between Raman signal of L-tyrosine and its concentration. The Ag nanoislands substrates were immersed in the aqueous solution of L-tyrosine at different concentrations (0.5-10 µM). The measurements were done with 100 µW power and 10 seconds acquisition time in liquid conditions (S8A Fig). Afterwards the curve areas values were calculated for the band centered at 1162 cm^-1^ (S8B Fig). S8C Fig shows the performed linear fit described by the following equation: y=a+bx (a=41000±4500; b=13700±1100).

S8 Fig. Calibration measurements for L-Tyrosine detection. (A) SERS spectra of L-Tyrosine at the concentrations 0.1-10 µM. (B) L-Tyrosine band at 1162 cm^-1^. (C) Linear fitting of 1162 cm^-1^ peak curve area versus concentration. The measurements were done with 532 nm laser line, 100 μW power and 10 s acquisition time in liquid conditions.
