# Supplementary material for: Time resolved and label free monitoring of extracellular metabolites by surface enhanced Raman spectroscopy
Source: PLoS One. 2017 Apr 18;12(4):e0175581. doi: 10.1371/journal.pone.0175581 (PMC5395151; doi:10.1371/journal.pone.0175581)
Supplement: S7 File — (DOCX) [file pone.0175581.s007.docx]

Supporting Information 7

Time resolved and label free monitoring of extracellular metabolites by surface-enhanced Raman spectroscopy

Victoria Shalabaeva^1^, Laura Lovato^1*^, Rosanna La Rocca^1^, Gabriele C. Messina^1^, Michele Dipalo^1^, Ermanno Miele^1^, Michela Perrone^1^, Francesco Gentile^2^, Francesco De Angelis^1*^

^1^ Plasmon Nanotechnologies, Istituto Italiano di Tecnologia, Genoa, Italy.

^2^ Department of Electrical Engineering and Information Technologies (DIETI), University Federico II of Naples, Naples, Italy.

^*^Corresponding authors:

E-mail:francesco.deangelis@iit.it (FDA); laura.lovato@iit.it (LL)

**Biocompatibility**

**Live/Dead Assay.** 2x10^5^ NIH/3T3 and Raw 264.7 cells were maintained in culture on the nanostructured Ag substrates for 48 hours and afterwards the viability was assessed with the LIVE/DEAD Viability/Cytotoxicity Kit, for mammalian cells (Thermo Fisher Scientific). The cells were incubated with Calcein AM 2 μM (4 mM stock solution) and 4 μM EthD-1 (2 mM stock solution) for 30 min at room temperature (RT) in the dark. The samples were analyzed with an inverted confocal microscope (A1 Nikon) using excitation wavelength of 488 nm for live-cells (green-fluorescent) and 530 nm for dead-cells (red-fluorescent). The images were acquired with 10x objective. The mean percentage of dead cells for the NIH/3T3 plated on the Ag nanoislands and for the control cultures on the glass coverslip after 2 DIVs was 11.13 ± 2.31 % and 7.68 ± 3.94 % respectively, as shown in S9A Fig, a and b. The mean percentage of dead cells for the Raw 264.7 plated on the Ag nanoislands and for the control cultures on the glass coverslip after 2 DIVs was 2.69 ± 0.85 % and 0.93 ± 0.44 %, respectively as shown in S9B Fig, a and b.

**S9 Fig. Live/dead assay.** Live/dead staining of NIH/3T3 (A) and Raw 264.7 (B) cells grown on Ag nanostructured substrates at 2 DIVs. (a) Black columns indicate the percentage of live cells, while the white columns represent the percentage of dead cells. ****p ≤ 0.0001 and ***p ≤ 0.0002. (b) The viability of NIH/3T3 and Raw 264.7 cells is evidenced by green live cells, in comparison to the red dead cells, on glass coverslips and Ag nanostructured substrates. Scale bar: 100 µm.

**Cells Fixation for SEM.** 2x10^5^ NIH/3T3 cells were plated on nanostructured Ag substrates and grown for 48 hours at 37ºC and 5% CO_2_. Afterwards the cells were fixed with glutaraldehyde 2% solution in deionized water for 40 min at RT. Then the cells were dehydrated with a series of 10 minutes incubations in rising concentrations of ethanol in water solutions (from 50% to 100%). Finally the samples were coated with a 20 nm thick Ag layer to perform SEM analysis.

**S10 Fig. SEM of cells.** SEM images of NIH/3T3 cells fixed at DIVs 2 on Ag island films. The cells are well-spread and show a flat morphology with well-visible filopodia. Scale bar: 20 µm.
