# Supplementary figures and images for: Time resolved and label free monitoring of extracellular metabolites by surface enhanced Raman spectroscopy
Source: PLoS One. 2017 Apr 18;12(4):e0175581. doi: 10.1371/journal.pone.0175581 (PMC5395151; doi:10.1371/journal.pone.0175581)

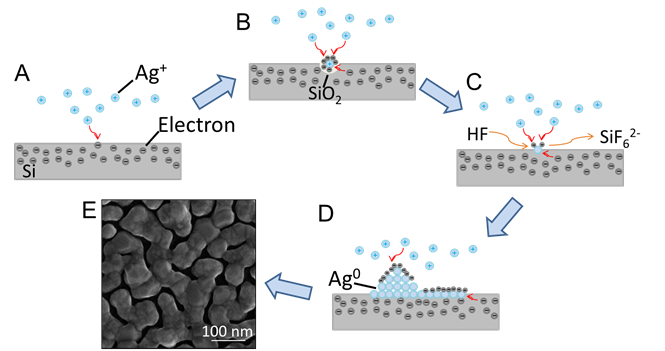

Supplement: S1 Fig — Scheme of the electroless deposition of Ag nanoparticles aggregates in water solution of AgNO3 and HF. (A) Redox reaction between Ag+ and Si: Ag+ ions in the vicinity with the silicon surface capture electrons from the valence band of Si. (B) Ag+ ions are reduced and deposited as metals while the silicon surface is oxidized into SiO2. (C) The redox reaction involves hydrofluoric acid, which induces the etching of SiO2 and the dissolution of SiF2−. (D) The Ag nuclei attract electrons from bulk silicon, become as a catalytic surface for the reduction of further Ag+ ions. (E) SEM images of a typical electroless grown Ag nanoislands pattern, the form and size of nanoislands is a function of the deposition time. (TIF) [file pone.0175581.s008.tif]

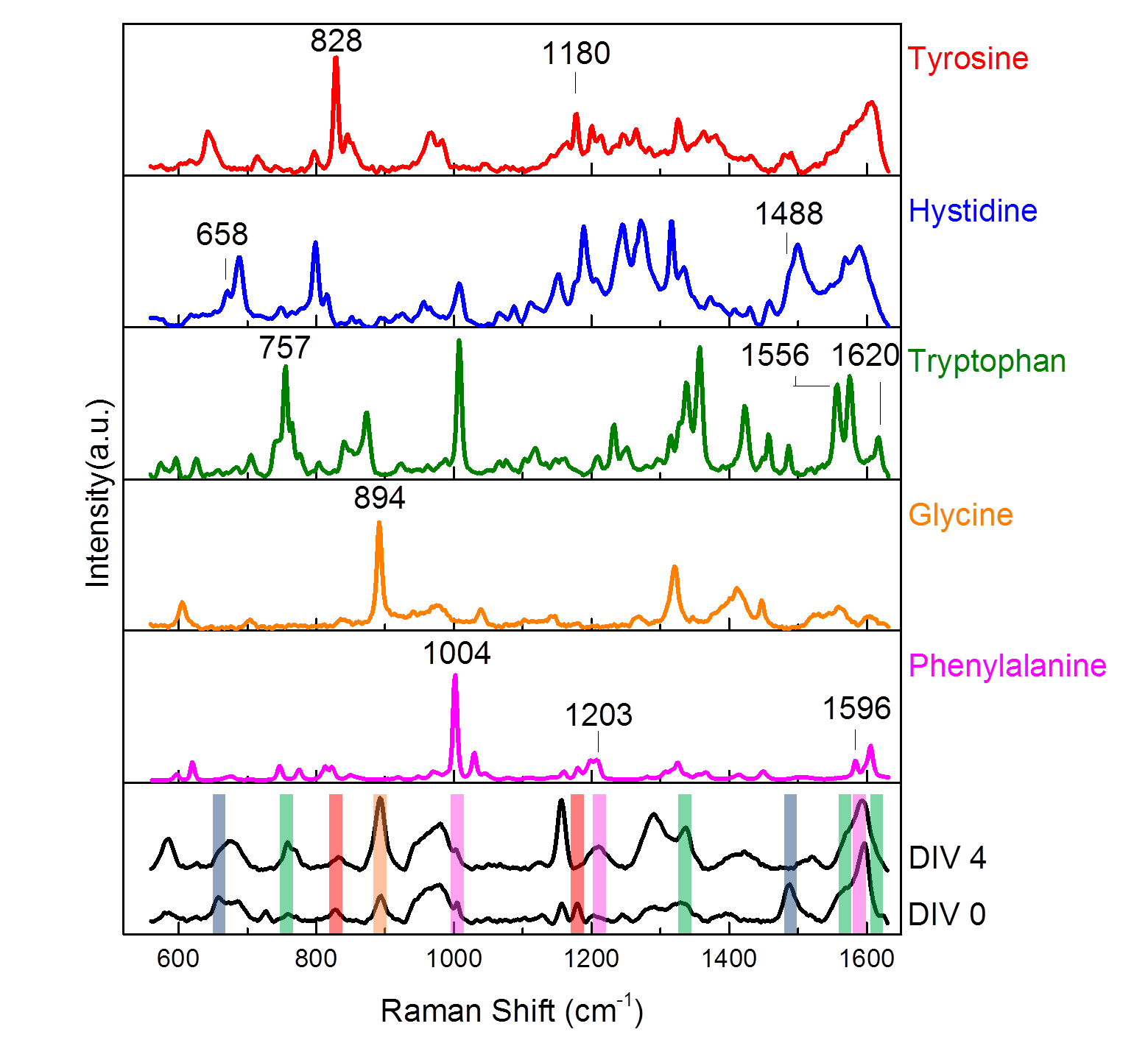

Supplement: S2 Fig — Raman spectra of the pure amino acids Tyrosine, Histidine, Tryptophane, Glycine, Phenylalanine are compared to the Raman spectra of the complete medium at DIV 0 and DIV 4. The characteristic peaks of each amino acid are highlighted in the medium spectra with different colors. (TIF) [file pone.0175581.s009.tif]

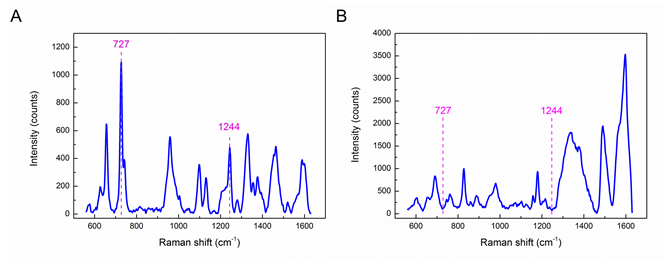

Supplement: S3 Fig — SERS spectrum of the separate cell medium components on the nanoislands Ag substrate: (A) Fetal bovine serum (FBS); (B) Dulbecco's modified Eagle's medium (DMEM) without red phenol. (TIF) [file pone.0175581.s010.tif]

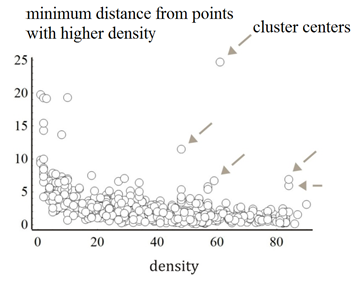

Supplement: S4 Fig — The diagram showing the cluster centers determination: cluster centers are the points in the set which have higher density respect to their neighbors and a relatively large distance from points with higher densities. (TIF) [file pone.0175581.s011.tif]

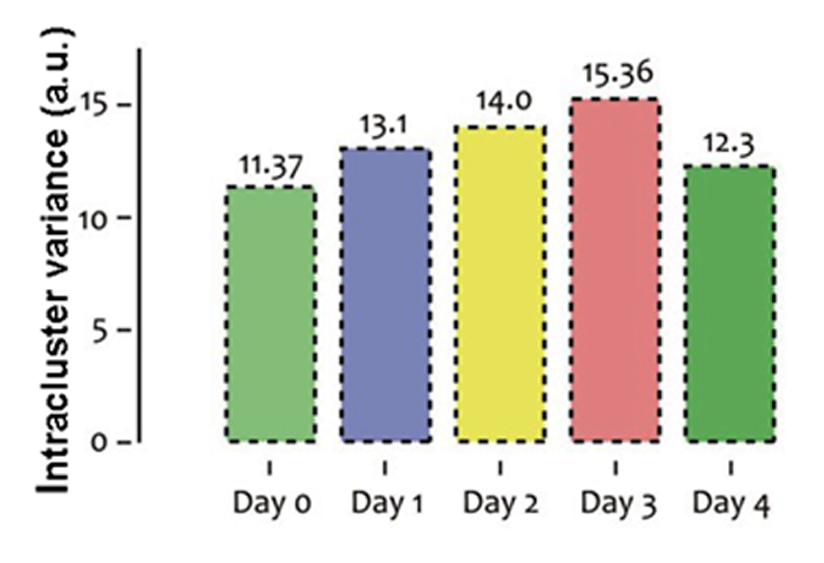

Supplement: S5 Fig — The variability within clusters presented in Fig 4B of the main text. (TIF) [file pone.0175581.s012.tif]

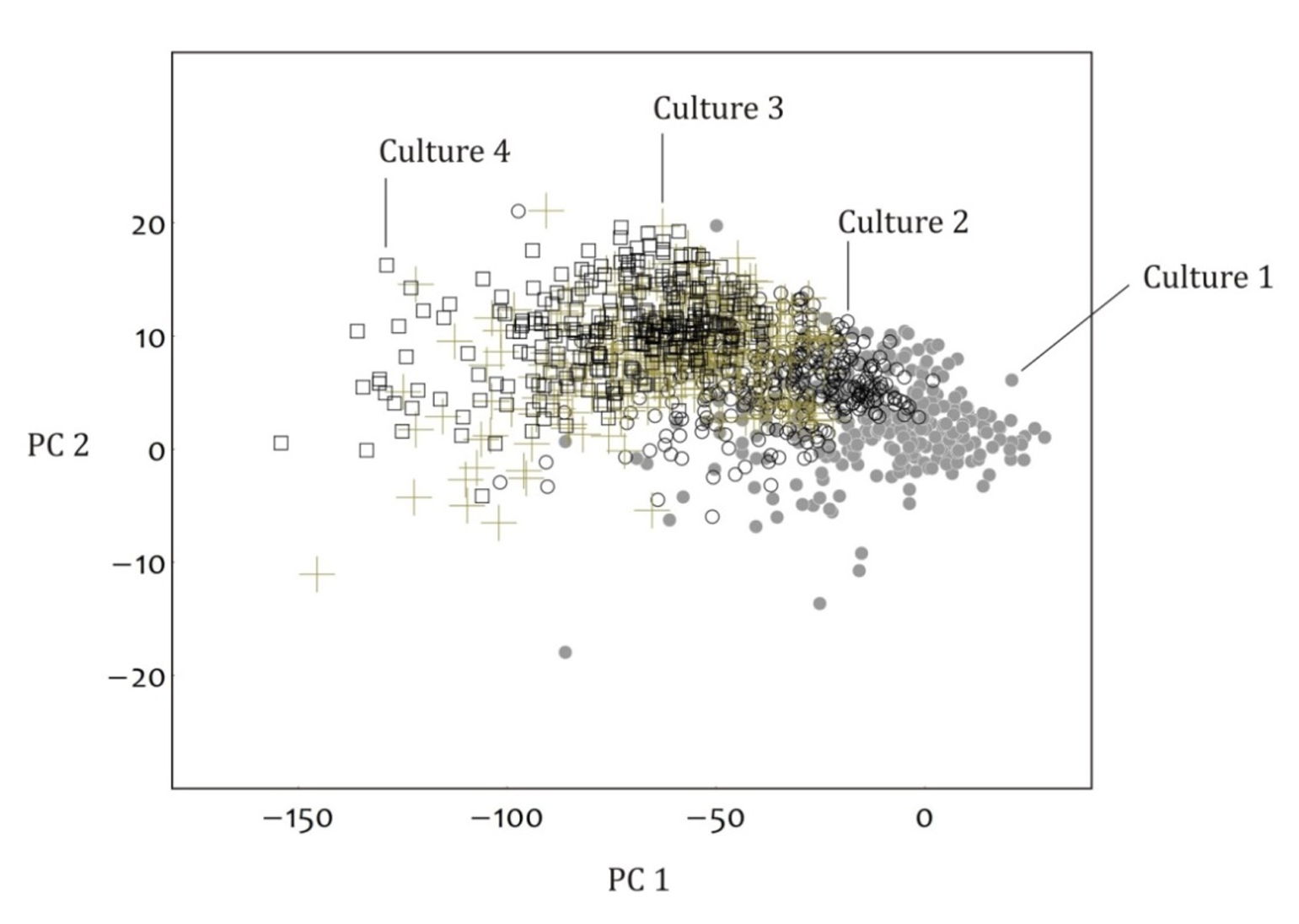

Supplement: S6 Fig — The PC1 vs PC2 scatter plot for different replicates across all the considered time points. (TIF) [file pone.0175581.s013.tif]

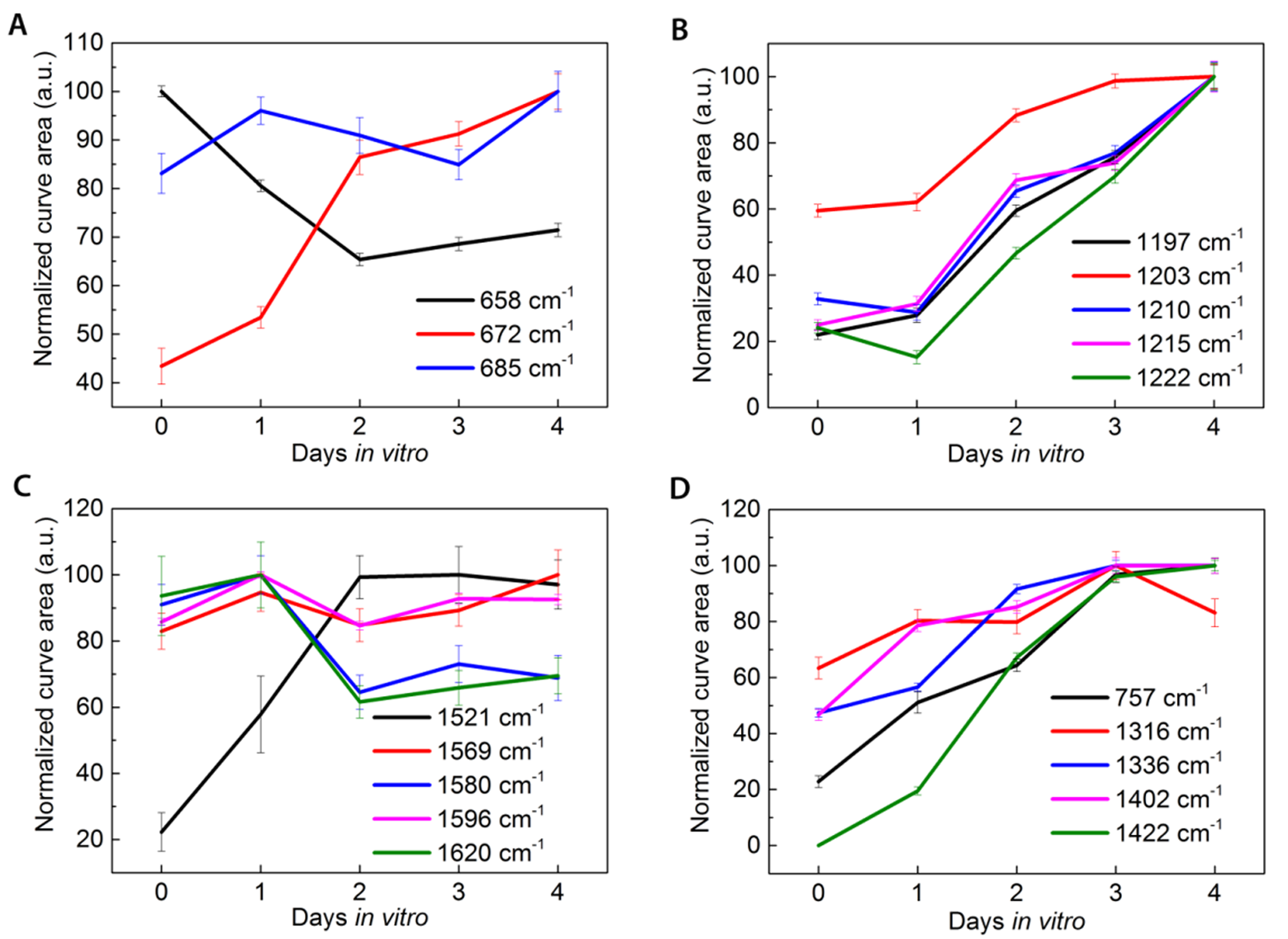

Supplement: S7 Fig — The rates of normalized integral curve areas of the peaks resolved in the spectral ranges: 640–716 cm-1 (A), 1190–1240 cm-1 (B), 1460–1629 cm-1 (C), 737–790 cm-1, 1250–1363 cm-1 and 1368–1460 cm-1 (D). (TIF) [file pone.0175581.s014.tif]

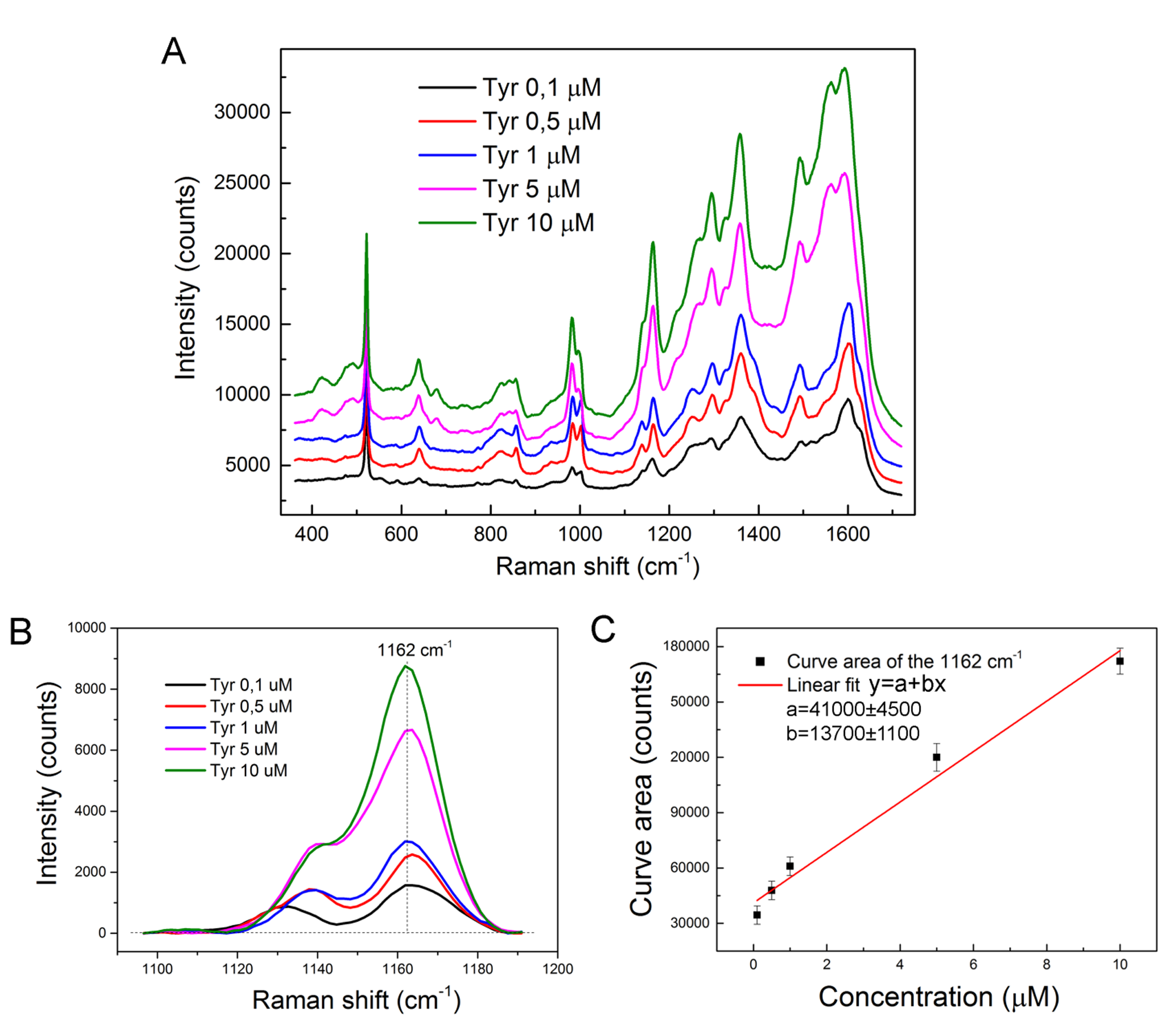

Supplement: S8 Fig — (A) SERS spectra of L-Tyrosine at the concentrations 0.1–10 μM. (B) L-Tyrosine band at 1162 cm-1. (C) Linear fitting of 1162 cm-1 peak curve area versus concentration. The measurements were done with 532 nm laser line, 100 μW power and 10 s acquisition time in liquid conditions. (TIF) [file pone.0175581.s015.tif]

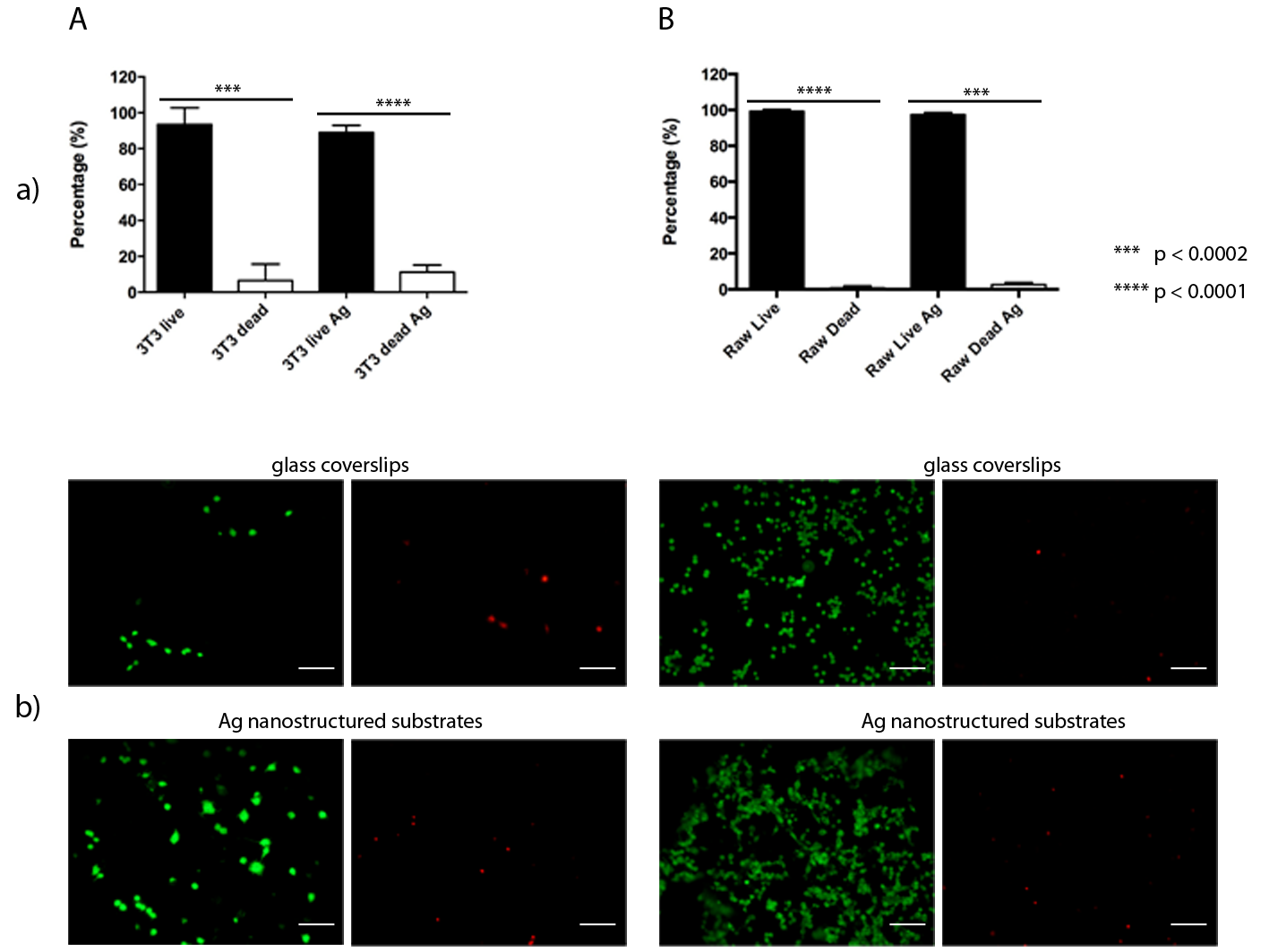

Supplement: S9 Fig — Live/dead staining of NIH/3T3 (A) and Raw 264.7 (B) cells grown on Ag nanostructured substrates at 2 DIVs. (a) Black columns indicate the percentage of live cells, while the white columns represent the percentage of dead cells. ****p ≤ 0.0001 and ***p ≤ 0.0002. (b) The viability of NIH/3T3 and Raw 264.7 cells is evidenced by green live cells, in comparison to the red dead cells, on glass coverslips and Ag nanostructured substrates. Scale bar: 100 μm. (TIF) [file pone.0175581.s016.tif]

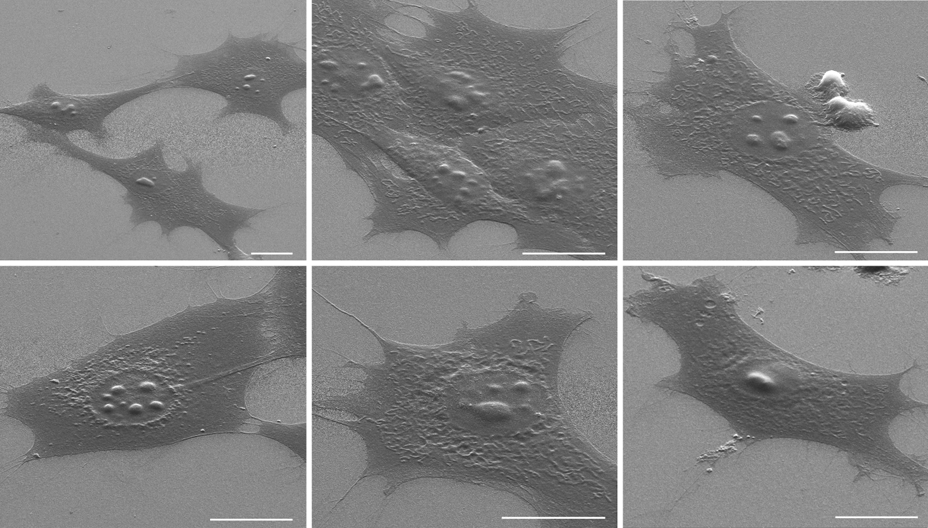

Supplement: S10 Fig — SEM images of NIH/3T3 cells fixed at DIVs 2 on Ag island films. The cells are well-spread and show a flat morphology with well-visible filopodia. Scale bar: 20 μm. (TIF) [file pone.0175581.s017.tif]
